# Efficacy of anti-PD-1 monotherapy for recurrent or metastatic olfactory neuroblastoma

**DOI:** 10.3389/fonc.2024.1379013

**Published:** 2024-05-23

**Authors:** Yuta Hoshi, Tomohiro Enokida, Shingo Tamura, Torahiko Nakashima, Susumu Okano, Takao Fujisawa, Masanobu Sato, Akihisa Wada, Hideki Tanaka, Naohiro Takeshita, Nobukazu Tanaka, Ryutaro Onaga, Takuma Kishida, Hideoki Uryu, Shingo Sakashita, Takahiro Asakage, Makoto Tahara

**Affiliations:** ^1^ Department of Head and Neck Medical Oncology, National Cancer Center East Hospital, Kashiwa, Japan; ^2^ Department of Head and Neck Surgery, Tokyo Medical and Dental University, Tokyo, Japan; ^3^ Department of Medical Oncology, National Hospital Organization Kyushu Medical Center, Fukuoka, Japan; ^4^ Department of Otolaryngology, Head and Neck Surgery, National Hospital Organization Kyushu Medical Center, Fukuoka, Japan; ^5^ Division of Pathology, Exploratory Oncology Research and Clinical Trial Center, National Cancer Center, Kashiwa, Japan

**Keywords:** olfactory neuroblastoma, immune checkpoint inhibitor, anti-PD-1 monotherapy, nivolumab, pembrolizumab

## Abstract

**Background:**

Olfactory neuroblastoma (ONB) is a rare malignant tumor of the head and neck. Due to its rarity, standard systemic therapy for this condition has yet to be established. In particular, the use of immune checkpoint inhibitors (ICIs) for the recurrent or metastatic (R/M) ONB population remains unclear.

**Methods:**

We retrospectively evaluated 11 patients with R/M ONB who received any systemic chemotherapy at two Japanese institutions (National Cancer Center Hospital East and Kyushu Medical Center) between January 2002 and March 2022 and analyzed outcomes by use of anti-PD-1 antibody (nivolumab or pembrolizumab) monotherapy.

**Results:**

Of the 11 patients, 6 received ICI (ICI-containing treatment group) and the remaining 5 were treated with systemic therapy but not including ICI (ICI-non-containing treatment group). Overall survival (OS) was significantly longer in the ICI-containing group (median OS: not reached *vs*. 6.4 months, log-rank *p*-value: 0.035). The fraction of ICI systemic therapy in the entire treatment period of this group reached 85.9%. Four patients (66.7%) in the ICI-containing treatment group experienced immune-related adverse events (irAE), with grades of 1/2. No irAE of grade 3 or more was seen, and no patient required interruption or discontinuation of treatment due to toxicity.

**Conclusion:**

ICI monotherapy appears to be effective and to contribute to prolonged survival in R/M ONB.

## Introduction

Olfactory neuroblastoma (ONB) is a rare tumor of the head and neck with an incidence of 0.4 per million, accounting for approximately 3% of all sinonasal tumors (1). It is a malignant neuroectodermal neoplasm with neuroblastic differentiation, often localized in the superior nasal cavity (2). For resectable disease, standard treatment is surgical resection followed by radiotherapy (3, 4). For locally advanced disease, induction chemotherapy followed by chemoradiotherapy improves local control and overall survival (OS) in some patients (5). In contrast, recurrent or metastatic ONB (R/M ONB) is an incurable condition, and the mainstay of treatment is palliative systemic therapy. Given the similarity of R/M ONB with small cell carcinoma or neuroendocrine carcinoma, treatment is often based around standard treatment for these latter conditions, namely cisplatin in combination with etoposide and other conventional cytotoxic drugs, including irinotecan, docetaxel, and vincristine (6, 7). Nevertheless, compared with the amount of evidence supporting the use of induction chemotherapy, little evidence is available for the use of anticancer drugs for R/M ONB.

Recently, immune checkpoint inhibitors (ICIs) have become available for several types of cancer, including recurrent or metastatic squamous cell carcinoma of the head and neck (R/M SCCHN) (8–10). In this patient population, the anti-programmed death 1 (PD-1) monoclonal antibodies nivolumab and pembrolizumab have been shown to provide a greater increase in OS than the conventional standard treatment. The US Food and Drug Administration consequently approved nivolumab for platinum-resistant R/M SCCHN and pembrolizumab for both platinum-resistant and -sensitive R/M SCCHN (11). In addition, our previous paper reported that nivolumab might help stabilize platinum-resistance ONB disease (12) Programmed cell death ligand 1 (PD-L1) expression level, determined by immunohistochemistry, is a clinically validated predictive biomarker of ICIs (13, 14). Although previous findings that PD-L1 expression in ONB tissue ranges from 0-40% (15, 16) suggest that ICIs might be effective for R/M ONB, the use of ICIs for these patients has not been reported and approved globally.

Here, we investigated the potential clinical utility of anti-PD-1 monotherapy for R/M ONB.

## Materials and methods

### Patients

We conducted a retrospective case series of 11 patients with R/M ONB who had received systemic therapy at two Japanese institutions (National Cancer Center Hospital East and Kyushu Medical Center) between January 2002 and March 2022. Cut-off date was the end of February 2023. Inclusion criteria were (1) histopathologically confirmed ONB; (2) presence of incurable local recurrence or distant metastasis; and (3) use of systemic therapy for R/M disease. Locoregional recurrence and distant metastasis were diagnosed by computed tomography (CT), magnetic resonance imaging (MRI), or positron emission tomography/computed tomography (PET-CT). We classified patients who had received ICI monotherapy for R/M disease as the ICI-containing treatment group and those who had never received ICI monotherapy for R/M disease as the other treatment group.

### Treatment

As no standard treatment for R/M ONB has been established, our patients are treated with cytotoxic anticancer drugs and molecular-targeting agents whose efficacy has been reported in case reports (5, 17, 18) or ICI monotherapy which may be applicable for at least stabilizing the disease of ONB (12) and usually associated with manageable toxicity. The types of ICI (pembrolizumab or nivolumab) used in the current study were determined based on platinum sensitivity according to the SCCHN. Concretely, nivolumab monotherapy was administered at 240 mg every two weeks to platinum-resistant patients (relapse within six months after last administration of a platinum agent). Dose and schedule modification to 480 mg every four weeks was allowed if tumor response was confirmed and the patient’s condition was stable. Pembrolizumab monotherapy was administered at 200 mg every three weeks to platinum-sensitive patients (relapse more than six months after last administration of a platinum agent). Nivolumab monotherapy or pembrolizumab monotherapy was continued until disease progression or intolerable toxicity, whichever occurred first. The other treatments included cetuximab (cetuximab 400 mg/m^2^ loading dose, then 250 mg/m^2^ per week) with or without chemotherapy (cisplatin 80 mg/m^2^ on day 1 and 5-fluorouracil 800 mg/m^2^ on days 1-4 administered every three weeks for up to 6 cycles, or paclitaxel 80 mg/m^2^ on day 1 every week) and cytotoxic chemotherapy without cetuximab (irinotecan 50-80 mg/m^2^ and docetaxel 30-50 mg/m^2^ on days 1, 8 and 15 every four weeks; cisplatin 80 mg/m^2^ on day 1, doxorubicin 40 mg/m^2^ on day 1 and etoposide 100 mg/m^2^ on days 1-3 every three weeks; carboplatin 5 AUC on day 1 and etoposide 80 mg/m^2^ on days 1-3 every four weeks; or tegafur gimeracil oteracil potassium 100 mg/m^2^ on days 1-14 every three weeks). We introduced ICI monotherapies for this patient population following the approval date for recurrent or metastatic head and neck carcinoma in Japan (March 2017 for nivolumab and February 2019 for pembrolizumab). Before approval, these patients had been treated exclusively with systemic therapies other than the ICIs mentioned above.

### Evaluation of efficacy and safety

Clinical tumor response to treatment was assessed radiographically using CT or MRI every 8-12 weeks, or sooner if the physician in charge deemed it necessary and the results were retrospectively evaluated according to Response Evaluation Criteria in Solid Tumors (RECIST) ver. 1.1. The diagnosis and evaluation of severity of immune-related adverse events (irAE) were based on clinical examinations and biological and imaging data.

### Statistical analysis

Progression-free survival (PFS) was defined as the time from first administration of nivolumab monotherapy or pembrolizumab monotherapy to the date of disease progression or death from any cause. OS was defined as the time from the first administration of systemic therapy to death from any cause. PFS and OS were calculated using the Kaplan-Meier method and evaluated using the log-rank test. The relationship between prognostic data and potential clinical prognostic factors was estimated by the Cox proportional hazards model or log-rank test. A P-value less than 5% was considered to indicate statistical significance. All statistical analyses were performed with EZR (version.1.51; Saitama Medical Center, Jichi Medical University, Saitama, Japan), a graphical user interface for R (The R Foundation for Statistical Computing, Vienna, Austria; version 4.1.1).

### PD-L1 expression and next-generation sequencing

PD-L1 expression was evaluated by PD-L1 IHC 22C3 pharmDx assay or PD-L1 IHC 28-8 pharmDx assay (Agilent Technologies, Carpinteria, CA, USA). Genomic profile testing (Foundation One® CDx) was used to assess genomic alteration in 324 genes, tumor mutation burden (TMB), and microsatellite instability (MSI) following DNA extraction from formalin-fixed, paraffin-embedded (FFPE) tissue specimens from either the primary tumor or lymph node metastasis.

## Results

### Patients and disease characteristics

A total of 11 patients with R/M ONB treated with systemic therapy during the study period were included. Baseline patient characteristics are shown in **Table 1**. Among all patients, six patients received ICI-containing treatment and five patients received other treatment without ICI. Baseline demographic and disease characteristics were generally well-balanced between the groups. Distant metastases tended to be slightly more common in ICI-non-containing treatment group. In all patients, distant metastatic sites included liver metastases in three cases, bone metastases and distant lymph node metastases in two cases each, and pleural dissemination, adrenal metastases and bone marrow metastases in one case each. None had central nerve system disease, such as leptomeningeal lesions. Details of treatment regimens are shown in **Supplementary Table S1**, and PD-L1 expression and tumor molecular profiling data in the ICI-containing treatment group are shown in **Supplementary Table S2**.

**Table 1:** Baseline characteristics of patients.

		All (N=11)	ICI-containing treatment group (n=6)	ICI-non-containing treatment group (n=5)	*p*-value^†^
**Median age**, years (range)^*^		48 (29-66)	50 (29-66)	48 (23-66)	0.783
**Gender**, n (%)	Male Female	6 (54.5) 5 (45.5)	4 (66.7) 2 (33.3)	2 (40.0) 3 (60.0)	0.782
**ECOG PS**, n (%)^*^	0/1 ≥ 2	9 (81.8) 2 (18.2)	6 (100) 0 (0)	3 (60.0) 2 (40.0)	0.354
**Modified Kadish**, n (%)^**^	A/B/C D	6 (54.5) 5 (45.5)	4 (66.7) 2 (33.3)	2 (40.0) 3 (60.0)	0.782
**Hyams’ Grade**, n (%)	I/II III/IV Unknown	6 (54.5) 3 (27.2) 2 (18.2)	4 (66.7) 2 (33.3) 0 (0)	2 (40.0) 1 (20.0) 2 (40.0)	0.231
**Initial curative therapy**, n (%)	Surgery alone Surgery followed by RT CRT alone IC followed by RT IC followed by CRT None	3 (27.2) 1 (9.1) 1 (9.1) 1 (9.1) 4 (36.4) 1 (9.1)	2 (33.3) 0 (0) 1 (16.7) 0 (0) 3 (50.0) 0 (0)	1 (20.0) 1 (20.0) 0 (0) 1 (20.0) 1 (20.0) 1 (20.0)	0.382
**Salvage surgery for locoregional lesion with curative intent before initiating systemic therapy**	Yes No	2 (18.2) 9 (81.8)	2 (33.3) 4 (66.6)	0 (0) 5 (100)	0.182
**Time from initial diagnosis to** **initiation of systemic therapy**, n (%)	< 1 year ≥ 1 year	5 (45.5) 6 (54.5)	3 (50.0) 3 (50.0)	2 (40.0) 3 (60.0)	1
**Local recurrence or** **distant metastasis** ^*^	Local recurrence alone Distant metastasis alone Both	7 (63.6) 2 (18.2) 2 (18.2)	5 (83.3) 0 (0) 1 (16.7)	2 (40.0) 2 (40.0) 1 (20.0)	0.164

^*^At recurrence or metastatic disease, ^**^At initial diagnosis. ^†^The t-test was used for continuous variables and the chi-squared test for categorical variables. ECOG PS, Eastern Cooperative Oncology Group Performance Status Scale; RT, radiotherapy; CRT, chemoradiotherapy; IC, induction-chemotherapy; and ICI, immune checkpoint inhibitor.

### Antitumor efficacy by ICI

In the ICI-containing treatment group, overall response rate (ORR) and disease control rate (DCR) by ICI were 33.3% (95% confidence interval [CI], 4.33-77.8) and 66.7% (95% CI, 22.3-95.7), respectively (**Table 2**). One patient (16.7%) was classified with complete response, 1 (16.7%) with partial response, 2 (33.3%) with stable disease, and 1 (16.7%) with progressive disease.

**Table 2:** Best overall response by ICI.

	CR	PR	SD	PD	NE	ORR^*^	DCR^**^	CBR^†^
**Nivolumab (n=4)**	1	1	0	1	1	50.0%	50.0%	50.0%
**Pembrolizumab (n=2)**	0	0	2	0	0	0%	100%	0%
**Total (n=6)**	1	1	2	1	1	33.3%	66.7%	33.3%

^*^Proportion of CR + PR. ^**^Proportion of CR + PR + SD. ^†^Proportion of CR + PR + SD at 6 months.

ICI, immune checkpoint inhibitor; CR, complete response; PR, partial response; SD, stable disease; PD, progressive disease; NE, not evaluable; ORR, objective response rate; DCR, disease control rate; and CBR, clinical benefit rate.

### Entire clinical course and prognosis

**Figure 1:**
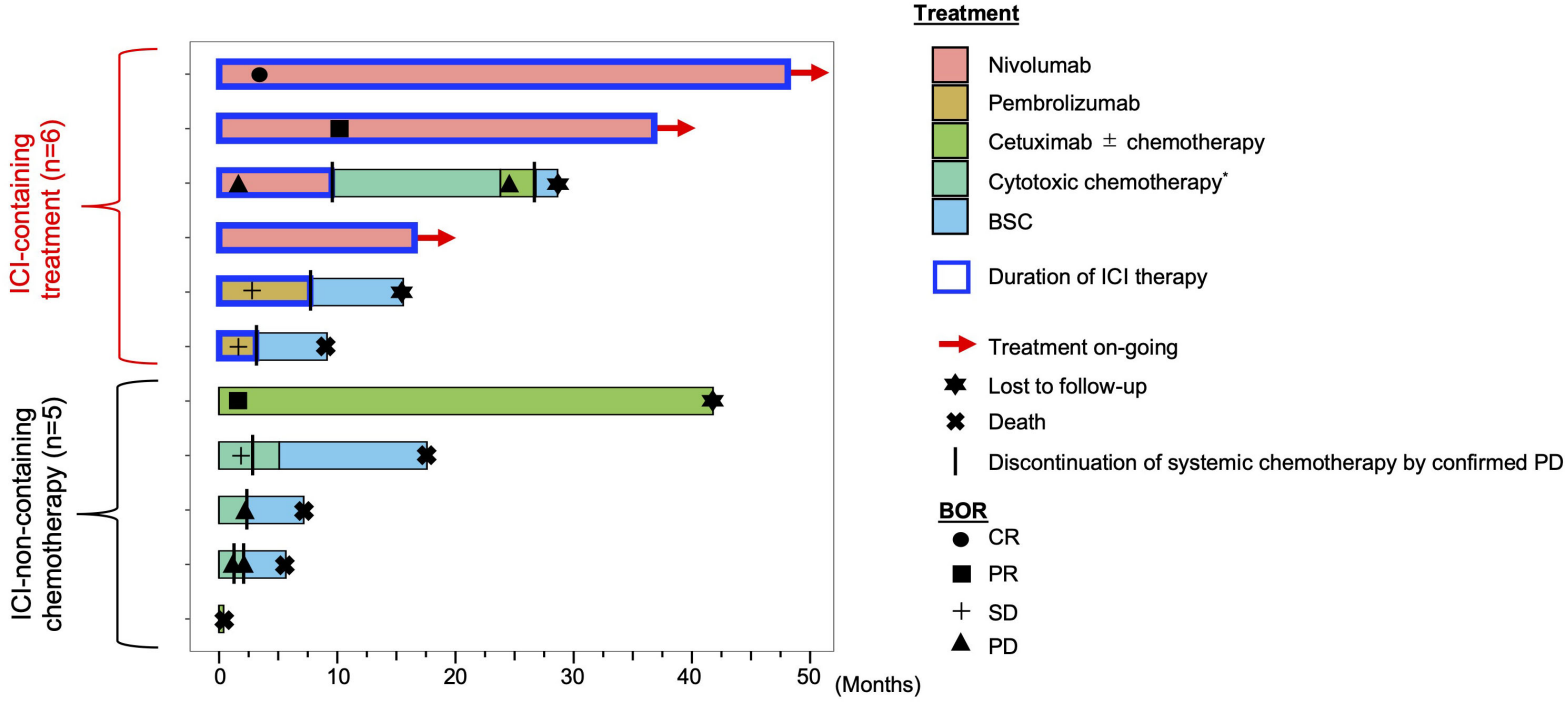
Swimmer plot of R/M ONB patients in the ICI-containing treatment group and other treatment group. ^*^cytotoxic chemotherapy is a systemic therapy, but does not include cetuximab. BOR, best overall response; CR, complete response; PR, partial response; SD, stable disease; PD, progressive disease; BSC, best supportive care; ICI, immune checkpoint inhibitor.

**Figure 1** shows the swimmer plot of all patients after the initiation of systemic chemotherapy for R/M disease in all cases. None had received palliative surgery or radiotherapy while anytime receiving systemic therapy. As of the cut-off date, three patients in the ICI-containing treatment group had been on nivolumab monotherapy for up to 48.6 months. The median duration of treatment with anti-PD-1 monotherapy was 13.8 months (range: 3.7-48.3), resulting in a median and average fraction of the anti-PD-1 monotherapy phase throughout the entire duration of systemic therapy reaching 100% and 85.9%, respectively (range: 16.5-100). With a median follow-up of 15.9 months, median PFS and 3-year PFS rate was 7.8 months (95% CI], 1.9-not reached [NR]) and 50.0% (95% CI, 11.1-80.4), respectively (**Supplementary Figure S1**). In the ICI-non-containing treatment group, one patient continued cetuximab-containing chemotherapy (platinum + 5-fluorouracil + cetuximab followed by cetuximab monotherapy) for up to 41.8 months, while duration under systemic therapy in this group was relatively shorter than that in the ICI-containing treatment group (median, 2.3 months *vs*. 13.2 months). **Supplementary Figure S2** shows representative images of the patient with CR after nivolumab administration.

**Figure 2:**
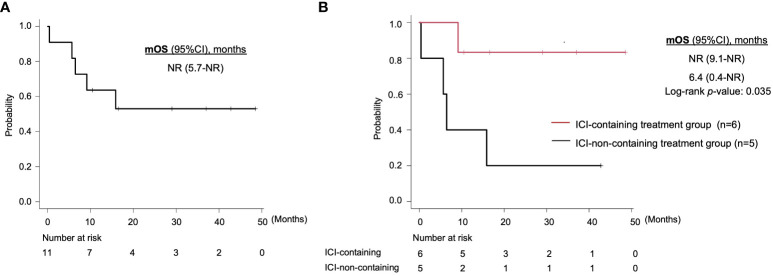
Kaplan-Meier curves of overall survival for **(A)** all patients and **(B)** the ICI-containing treatment group and other treatment group. mOS, median overall survival; ICI, immune checkpoint inhibitor; CI, confidence interval; NR, not reached.

With regard to survival, median OS for all patients was not reached (95% CI, 5.7-NR), and 1-year OS rate was 63.6% (95% CI, 29.7-84.5) (**Figure 2A**). On comparison, the ICI-containing treatment group had a significantly longer OS (median OS: NR *vs*. 6.4 months, 1-year OS: 83.3% (95%CI, 27.3-97.5) *vs*. 40.0% (95% CI, 5.2-75.3), log-rank *p*-value: 0.035) (**Figure 2B**). On univariate analysis, ECOG PS (hazard ratio [HR], 4.89; 95% CI, 1.14-21.01; *p* = 0.03) and distant metastasis (HR, not evaluable; *p* = 0.0004) were associated with OS (**Table 3**). The use of ICI, assessed by the Cox proportional hazards model, also tended to be associated with better OS (HR, 0.13; 95% CI, 0.01-1.20; *p* =0.07).

**Table 3:** Univariate analysis for overall survival.

Variable	Category	Number of patients (%)	HR (95% CI)	*p*-value
**Age**, years	< 65 ≥ 65	9 (81.8) 2 (18.2)	Reference 1.84 (0.19-17.99)	0.60
**Gender**	Male Female	6 (54.5) 5 (45.5)	Reference 0.80 (0.13-4.81)	0.81
**ECOG PS**	0/1 ≥ 2	9 (81.8) 2 (18.2)	Reference 4.89 (1.14-21.01)	0.03
**Modified Kadish**	A/B/C D	6 (54.5) 5 (45.5)	Reference 0.87 (0.14-5.22)	0.87
**Hyams’ Grade**	I/II III/IV	6 (54.5) 3 (27.2)	Reference 0.77 (0.08-7.49)	0.83
**Distant metastasis**	No Yes	7 (63.6) 4 (36.4)	Reference NE	0.0004^*^
**Use of ICI**	No Yes	5 (45.5) 6 (54.5)	Reference 0.13 (0.01-1.20)	0.07

^*^The log-rank test was used instead of the Cox proportional hazards model because no events occurred in one of the groups. ECOG PS, Eastern Cooperative Oncology Group Performance Status Scale; ICI, immune checkpoint inhibitor; CI, confidence interval; and NE, not evaluable.

### Safety profile by ICI

irAEs caused by anti-PD-1 monotherapy are shown in **Table 4**. Four patients (66.7%) experienced adverse events of any grade, among which pruritus, adrenal insufficiency, liver dysfunction, fatigue, and hypothyroidism were common. No patient developed an adverse event of grade 3 or higher or had treatment interrupted or discontinued due to an irAE.

**Table 4:** Immune-related adverse events by ICI.

	Number of patients (n = 6) (%)	
	Any Grade	≥ Grade 3
**Pruritus**	4 (66.7)	0 (0)
**Adrenal insufficiency**	2 (33.3)	0 (0)
**ALT increased**	2 (33.3)	0 (0)
**AST increased**	2 (33.3)	0 (0)
**Fatigue**	2 (33.3)	0 (0)
**Hypothyroidism**	2 (33.3)	0 (0)
**Anorexia**	1 (16.7)	0 (0)
**Diarrhea**	1 (16.7)	0 (0)
**Rash**	1 (16.7)	0 (0)
**Total**	4 (66.7)	0 (0)

Graded according to Common Toxicity Criteria for Adverse Events version 5.0.

ALT, alanine aminotransferase; AST, aspartate transaminase.

## Discussion

In this study, we found that anti-PD-1 monotherapy induced a tumor response in patients with R/M ONB and provided disease control, resulting in an objective prolongation of survival compared with patients who did not receive one of these agents in their clinical course. No new safety concerns were identified. To our knowledge, this is the first case series to assess the clinical efficacy of anti-PD-1 monotherapy and its potential effect on survival in R/M ONB patients.

Although ONB patients with non-distant disease are treated curatively, 12% develop distant metastatic disease (19). In addition, 8% of ONB patients present with distant metastasis at initial diagnosis (20). These R/M ONB patients are generally challenging to treat radically and usually receive palliative systemic chemotherapy. However, the significance of platinum-based cytotoxic chemotherapy for ONB patients with distant metastasis has not been demonstrated by systemic review or meta-analysis (19). For molecular targeted therapy, several drugs (e.g., sunitinib, everolimus, and pazopanib) have shown relatively favorable disease control in patients harboring a corresponding genetic alteration (21, 22), but most patients do not have these targets and barely benefit from this treatment. Although peptide receptor radionuclide therapy (PRRP), which is based on the relatively high positivity of somatostatin receptor 2 (SSTR2), has been under evaluation (23, 24), therapeutic options for this patient population remain limited. Against this background, our present findings – an ORR of 33.3% and long-term disease control for up to 48.6 months in some patients – may make ICI monotherapy one of the therapeutic options for R/M ONB. Furthermore, we saw no new safety concerns regarding irAE in terms of either incidence or severity (any grade: 66.7%, grade 3/4: 0%) on comparison with reports obtained from a systematic review of toxicity with ICI monotherapy (any grade: 54-76%, grade 3/4: 28-72%) (25).

Allowing that the number of enrolled patients was small, we attempted to evaluate both clinical factors as well as biomarkers to identify patient populations more likely to benefit from ICI monotherapy. With regard to clinical factors, general reports in cancer patients, including those with ONB (26, 27), indicate that the maintenance of ECOG PS (PS 0/1) and absence of distant metastasis were associated with favorable prognosis in the current study (**Table 3**). Patients meeting these criteria might be suitable candidates for therapy. With regard to biomarkers, although we cannot exclude the possibility of temporal and spatial variation in PD-L1 expression, no apparent association with ICI efficacy was observed in the current study (two responders both had PD-L1-negative disease; **Supplementary Table S2**), despite a generally positive correlation between PD-L1 expression and response to ICI as well as survival prognosis in other types of cancer (13, 28, 29). Further, TMB and MSI status also appeared not to correlate with the clinical efficacy of ICI (one evaluated responder had 0/mb TMB; **Supplementary Table S2**). Again, although the small number of evaluable subjects hampers the reaching of conclusive results, these markers would not be definitive in eliminating the clinical application of ICI monotherapy in R/M ONB. At the same time, further exploration to identify additional biomarkers is warranted.

Several limitations of our study should be mentioned. First, the study was conducted under a retrospective case series with a small number of patients, as mentioned above. We cannot exclude the possibility of selection bias in survival prognosis on comparison with the ICI-non-containing treatment group; for example, distant metastasis was more frequent in the latter group (16.7% *vs*. 80.0%, *p*=0.391, **Table 1**). Prospective evaluation in a larger number of patients is required to confirm our findings. Second, consistent with general discussion in this field, we have no plausible insight into the validity of continuing ICI monotherapy in patients who achieve a long-term tumor response or stabilization. Three subjects are still under ICI monotherapy as their treatment-related AEs are manageable (limited to hormone replacement therapy for immune-related hypothyroidism and adrenal insufficiency). A previous clinical trial reported that non-small-cell lung cancer (NSCLC) patients exhibited a positive tumor response to one year of nivolumab monotherapy, and found that subjects who continued nivolumab monotherapy showed significant improvements in both PFS and OS compared to those who discontinued this monotherapy (30). Other recent retrospective studies have reported that NSCLC patients who continued for more than two years had no difference in time to treatment failure compared with those who discontinued after two years and an increase in irAE in the continued ICI group 5(31). Given these past and present findings, and the currently limited alternative treatment options, we consider that continuation of ICI monotherapy for R/M ONB responders is reasonable. On the other hand, discontinuation of ICI is also an option, especially in the subject with the stabilized disease for a long time or with intolerable irAE.”

## Conclusion

We demonstrated that anti-PD-1 monotherapy is a safe and effective treatment for R/M ONB. The treatment may be a therapeutic option for this patient population, whose treatment strategies remain limited and not standardized.

## Data availability statement

The datasets presented in this study can be found in online repositories. The names of the repository/repositories and accession number(s) can be found in the article/**Supplementary Material**.

## Ethics statement

The studies involving humans were approved by the Institutional Ethical Committee of the National Cancer Center Hospital East. The studies were conducted in accordance with the local legislation and institutional requirements. The participants provided their written informed consent to participate in this study.

## Author contributions

YH: Writing – original draft, Data curation, Conceptualization. TE: Writing – review & editing. ST: Writing – review & editing, Data curation. TN: Writing – review & editing. SO: Writing – review & editing. TF: Writing – review & editing. MS: Writing – review & editing. AW: Writing – review & editing. HT: Writing – review & editing. NaT: Writing – review & editing. NoT: Writing – review & editing. RO: Writing – review & editing. TK: Writing – review & editing. HU: Writing – review & editing. SS: Writing – review & editing. TA: Writing – review & editing, Supervision. MT: Writing – review & editing, Supervision.
