# Supplementary material for: Efficacy of anti-PD-1 monotherapy for recurrent or metastatic olfactory neuroblastoma
Source: Front Oncol. 2024 May 23;14:1379013. doi: 10.3389/fonc.2024.1379013 (PMC11153745; doi:10.3389/fonc.2024.1379013)
Supplement: Supplementary file 1 [file DataSheet_1.docx]

# Supplementary Figures and Tables

**Supplementary Figure1.** Kaplan-Meier curves of progression-free survival for patients treated with nivolumab monotherapy or pembrolizumab monotherapy.


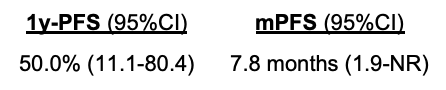


(Months)

Probability

1.0

0.8

0..6

0.4

0.0

0.2

0

10

30

40

50

20

Number at risk

6

3

2

1

0

2

Abbreviations: 3-y PFS, three-year progression-free survival; mPFS, median progression-free survival; ICI, immune checkpoint inhibitor; CI, confidence interval; NR, not reached.

**Supplementary Figure 2.** Representative images of an incurable recurrent patient who achieved complete response by nivolumab monotherapy

Initial diagnosis

as an incurable recurrent olfactory neuroblastoma

38.5 months after

nivolumab administration


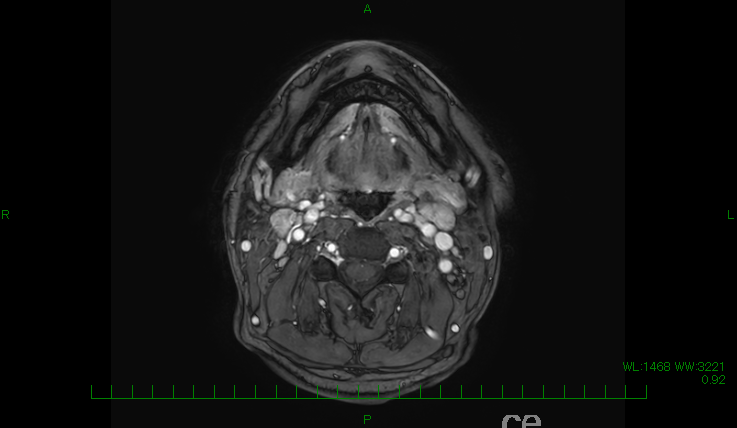

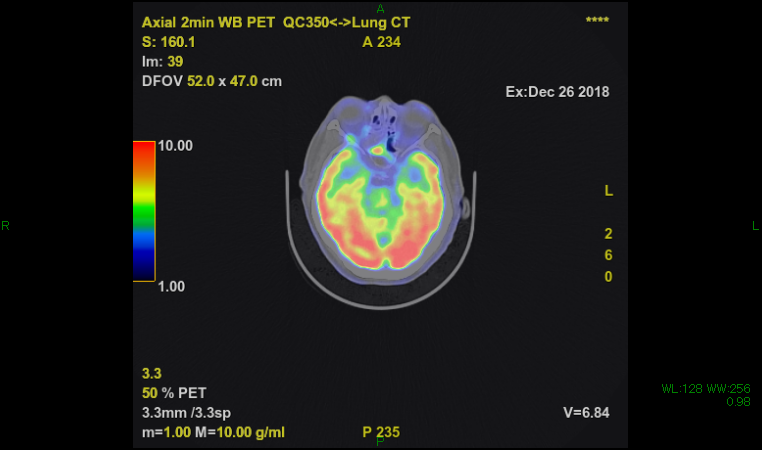

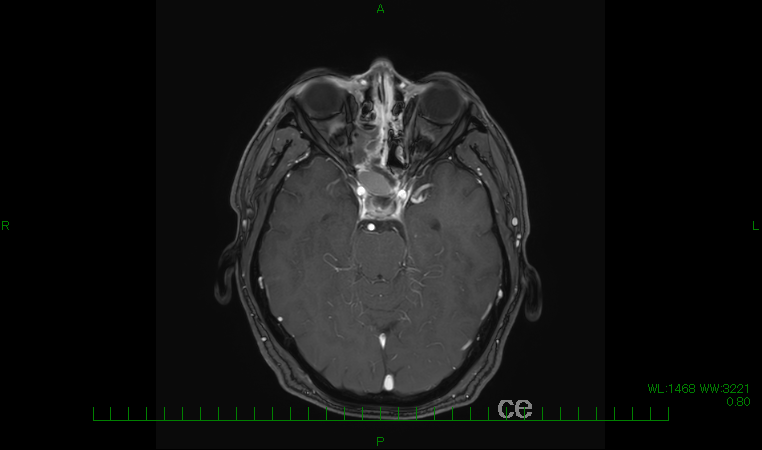

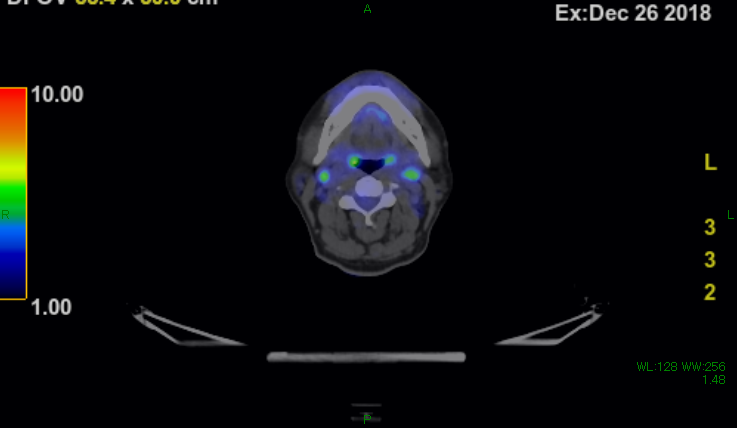

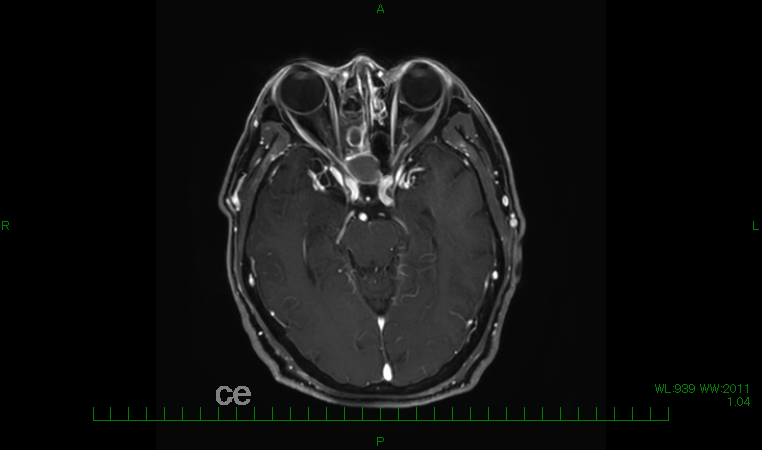

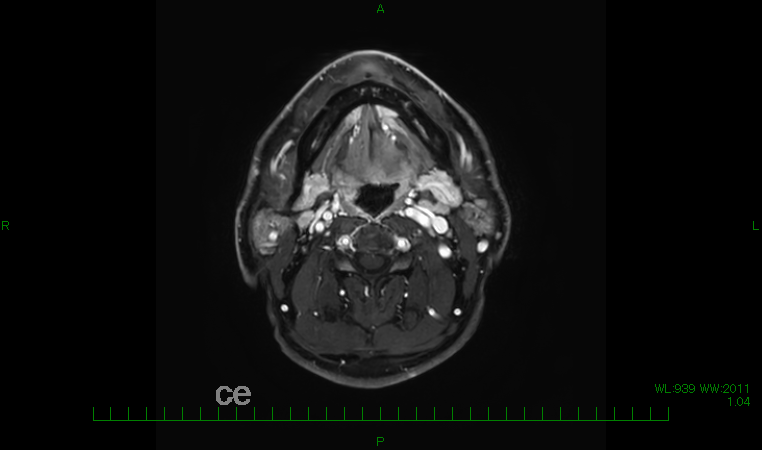


A

D

B

C

F

E

**A-D**. Magnetic resonance imaging (MRI) (**A, B**) and positron emission tomography-CT (PET-CT) (**C, D**) show primary residual lesion and cervical lymph node metastasis with the uptake of 18FDG uptake (arrows). **E, F**. MRI shows the disappearance of the primary residual lesion and shrinking of cervical lymph node metastasis to the normal size (arrows) at 38.5 months after initiation of nivolumab monotherapy.

**Supplementary Table S1.** Systemic therapy in each group

| **Group** | **Regimen**^*^ | **Number of patients** (%) |
| --- | --- | --- |
| **ICI-containing treatment (n=6)** | Nivolumab  Pembrolizumab  CBDCA + ETP  Cmab + PTX^**^ | 4 (66.7)  2 (33.3)  1 (16.7)  1 (16.7) |
| **ICI-non-containing treatment (n=5)** | Cmab^**^  CDDP + DXR + ETP  DTX + IRI  Cmab + PF^**^  S-1 | 1 (20.0)  1 (20.0)  3 (60.0)  1 (20.0)  1 (20.0) |

^*^Some overlap present due to multiple treatment in some patients. ^**^ Categorized as “cetuximab ± chemotherapy” in Figure 1. Abbreviations: BSC, best supportive care; CBDCA, carboplatin; CDDP, cisplatin; Cmab, cetuximab; DTX, docetaxel; DXR, doxorubicin; ETP, etoposide; PF, cisplatin and 5-fluorouracil; ICI, immune checkpoint inhibitor; IRI, irinotecan; PTX, paclitaxel; and S-1, tegafur gimeracil oteracil potassium.

**Supplementary Table S2.** Expression of PD-L1 and genomic profiling in patients treated with ICI

| **Case** | **Regimen** | **BOR of ICI** | **Hyams’ grade** | **Local or distant**  **recurrent site** | **PD-L1  expression** | **NGS** | **TMB [/mb]** | **Pathogenic genomic alteration** | **MSI** |
| --- | --- | --- | --- | --- | --- | --- | --- | --- | --- |
| **Case 1** | Nivolumab | CR | Ⅲ | Primary site  Regional lymph node | TPS = 0 | NE | - | - | - |
| **Case 2** | Nivolumab | PR | III | Regional lymph node | TPS = 0 | Done | 0 | NF2 E594^＊^ | MSS |
| **Case 3** | Nivolumab | PD | Ⅰ | Primary site | NE | Done | 1 | Not identified | MSS |
| **Case 4** | Nivolumab | NE | Ⅱ | Primary site  Regional lymph node | TPS < 1 | Done | 1 | CCND1 amplification;  FGF19 amplification;  FGF4 amplification;  NTRK1 R593W | MSS |
| **Case 5** | Pembrolizumab | SD | Ⅰ | Primary site  Regional lymph node | CPS = 0 | Done | 4 | ARID1A Y915^＊^;  PBRM1 E1103^＊^ | MSS |
| **Case 6** | Pembrolizumab | SD | I | Primary site  Regional lymph node  Distant lymph node  Bone  Pleural dissemination | CPS = 0 | NE | - | - | - |

Abbreviations: BOR, best overall response; CR, complete response; PR, partial response; SD, stable disease; PD, progressive disease; TPS, tumor proportion score; CPS, combined positive score; NGS, next generation sequencing; TMB, tumor mutation burden; MSI, microsatellite instability; MSS, microsatellite stable; and NE, not evaluable.
